# Pediatric effects of forever chemicals may last for a long time

**DOI:** 10.1038/s41390-026-05366-w

**Published:** 2026-10-07

**Authors:** Philippe Grandjean

**Affiliations:** 1 National Institute of Public Health, University of Southern Denmark, Copenhagen, Denmark; 2 Department of Biomedical and Pharmaceutical Sciences, University of Rhode Island, Kingston, RI USA

## Abstract

**Abstract:**

Perfluorinated alkylate substances (PFASs) have been in use for about 75 years. Because of their persistence, concentrations in the environment have increased, and absorbed PFAS will remain in the human body, some compounds for many years. The fetus and the newborn infant are particularly vulnerable to PFAS toxicity due to the intense development of organs and body functions. In addition, PFASs pass the placenta so that a pregnant woman shares her accumulated PFAS burden with the progeny. In addition, PFASs are excreted in the milk, so that the resulting serum-PFAS concentration in the infant may greatly exceed that of the mother. Multi-organ effects have been reported, some related to endocrine disruption. Immunotoxicity can occur at very low exposure levels, with elevated risks of infectious disease and possibly also cancer development. Unfortunately, little research was carried out during the first 50 years of industrial use, and some study reports were hidden. Independent research has been carried out only recently. Due to incomplete evidence, governmental regulations have been delayed and remain insufficient, in part because of objections from industrial interests.

**Impact:**

The perfluorinated alkylate substances (PFASs) are persistent and accumulate in the environment and in the human body. The greatest risk of PFAS toxicity occurs at early age, as PFASs are transferred through the placenta and human milk. This means that early-life exposures may trigger development of diseases and other adverse outcomes that may seriously burden adult health. Research has been delayed due to secrecy. While pediatric studies in this field are underway, prudent decisions to protect public health should be made despite possible concerns regarding incomplete evidence.

Practical complications, and current guidelines and interventions are by far insufficient.

- Current evidence on PFASs shows that these industrial chemicals are persistent and that highly elevated exposures occur already at early age.
- The maternal body burden of PFAS is shared with the fetus, and substantial amounts are transferred in human milk, thus impacting the unique nutritional advantage of breast-feeding.
- Recent evidence suggests that early PFAS exposure can affect public health, also during adult age, and that some adverse effects may be transferred to the next generation.

## Introduction

The perfluoroalkyl substances (PFASs) do not occur naturally. Rather, PFASs constitute a large, complex, and ever-expanding class of synthetic chemicals.^1^ Like alkanes, PFASs have a carbon “backbone”, but they are characterized by the replacement of hydrogen (H) atoms with fluorine (F) atoms that are bonded to the carbon backbone. The bond between carbon and fluorine is one of the strongest in chemistry. PFASs therefore show high thermal, chemical, and biological inertness; properties that make them useful for industrial purposes that benefit from PFASs continually repelling oil, gas, and water.^1,2^ Accordingly, PFASs are used and have been used in a broad host of commercial and consumer products including as a surface protectant, coating on food packaging, and in aqueous film-forming foams (AFFF) used for firefighting.^1,2^

Yet, the same properties that make these chemicals attractive for industrial use also form the basis for PFASs posing a risk to the environment and to human health, thus being known as “forever chemicals”.^3^ The major PFASs are known to be virtually indestructible in the environment and in the human body, thus resulting in persistence and bioaccumulation.^1^ In the beginning, the PFASs most commonly used were perfluorooctanoic acid (PFOA) and perfluorooctanesulfonic acid (PFOS).^4,5^ The use of these specific PFASs was largely discontinued after their harmful effects to human health eventually caused concern in the early years of this millennium.^1^ Thus, in 2006, the U.S.EPA invited eight major PFAS producers to join the PFOA Stewardship Program, which pledged to reduce 95% of their facilities’ PFOA emissions by 2010.^6^

As a result of environmental releases over the years, PFASs are frequently detected in food, most commonly in fish and shellfish,^7^ and intake of seafood and freshwater fish therefore predicts higher blood concentrations of PFASs.^8–10^ In a review of food-mediated exposures, eggs, vegetables, and venison were also emphasized as common sources of PFAS exposure in the EU,^7^ and available data from the U.S seem to agree with this conclusion.

Studies confirm that PFASs are easily absorbed once ingested from contaminated sources and through inhalation of contaminated air.^1^ Much less is absorbed through the skin, unless applied with substances that act as carriers.^11^ The long-chain PFASs are particularly persistent in the human body and therefore stay in the blood and in organs for a very long time.^1^ This means that continuing exposure results in accumulation of the PFASs in the body, where they will remain for a long time after cessation of exposure.

From serial analyses of serum samples from former 3 M production workers after retirement, biological half-lives (T½) for long-chain PFASs have been estimated to be ~3 years (PFOA), ~5 years (PFOS), while most short-chain PFASs have a shorter T½ in serum.^12^ However, the T½ for perfluorohexane sulfonic acid (PFHxS) is even longer, i.e., ~ 9 years. The T½ appears to be slightly less than 2.5 years for perfluorononanoic acid (PFNA).^13,14^ If the exposure is not completely eliminated, the body burden will decrease more slowly (if at all), thus leading to a longer apparent T½.^15^ All of these PFASs will therefore accumulate and remain in the body for many years.

## Adverse effects in early life

The 2022 report from the National Academy of Sciences, Engineering and Medicine (NASEM)^16^ found sufficient evidence for the following four adverse effects: infant and fetal growth, antibody response (in adults and children), dyslipidemia (elevated serum-cholesterol, in adults and children), and kidney cancer. Among the outcomes with only suggestive evidence, NASEM mentioned breast cancer (better information is now available, see below). Insufficient evidence was available at the time for, e.g., diabetes, obesity, metabolic syndrome, cardiovascular disease, reproductive toxicity, and additional types of cancer, according to the NASEM review. More recent evidence has added to the likelihood of these adverse effects being of concern and that early-life exposures may be particularly harmful.^17^ Major assessments over time have shown a general tendency for the scientific evidence of PFAS toxicity to become stronger (Table 1). These adverse effects are of special relevance in regard to developmental PFAS exposures.

**Table 1:** Adverse effects considered possibly linked to PFOA exposure by the C8 investigations,^64^ associations with PFAS exposure listed by the NASEM^16^ 10 years later, and as currently reviewed.

	C8 link to PFOA (C8) exposure	NASEM evidence of PFAS association	My assessment
Antibody response (immunotoxicity)	-	Sufficient	Sufficient
Dyslipidemia (high cholesterol)	Probable	Sufficient	Sufficient
Infant and fetal growth	-	Sufficient	Sufficient
Pregnancy-induced hypertension/preeclampsia	Probable	Suggestive	Sufficient
Thyroid disease	Probable	Suggestive	Suggestive
Ulcerative colitis	Probable	Suggestive	Possible
Liver enzyme alterations	-	Suggestive	Sufficient
Kidney cancer	Probable	Sufficient	Sufficient

### Reproduction

In respect to human reproductive system functions, PFAS exposure is associated with a broad range of adverse effects on reproduction documented in adult women and men. For women, PFAS exposure can lead to higher rates of infertility, miscarriage, pre-eclampsia and higher blood pressure during pregnancy.^18–21^

A reduced chance of becoming pregnant was found in Canada for elevated serum concentrations of both PFOA and PFHxS.^22^ PFAS-associated development of endometriosis may play a role in this regard.^23^ A Chinese study identified perfluoroheptanoic acid (PFHpA) exposure as a risk factor for decreased couple fecundity,^24^ as also seen in reports on pregnancy success after in vitro fertilization.^25,26^ Although effects at lower exposure levels may be weaker,^27^ the abnormalities identified may well interfere with a woman’s ability to have a safe and healthy pregnancy and delivery. In vitro fertilization appears to be less successful at higher parental PFAS exposures.^25,26^ A causal connection is likely, as demonstrated by experimental studies and elucidation of potential mechanisms,^28^ although earlier epidemiological studies seemed to show less clear associations.^29^

In men, PFAS exposure can result in lower sperm counts and fertility rates,^30–32^ and that may be related to exposures earlier in life.^33^ A joint analysis of data from three countries suggested a substantially lower proportion of morphologically normal sperm cells at increased serum concentrations of PFOA and PFHxS.^31^

The most extensive documentation shows that prenatal exposure to PFASs can result in preterm birth, low birth weight, and delayed fetal growth,^34–37^ as also highlighted by agency reviews.^1,38,39^ The most recent study from a contaminated area in Italy also showed increased numbers of infants born small for gestational age, as well as increased occurrence of low birth weight.^40^ In later childhood, elevated prenatal PFAS exposures may lead to increased body weight and body mass index,^41,42^ as has recently been shown in a meta-analysis.^43^

Despite early observations of possible PFAS-associated malformations, little research has been carried out to assess abnormalities of birth outcomes beyond decreased birth weight.^16^ A population study from China suggested that PFAS exposure may result in an increased occurrence of congenital heart defects,^44^ and one study examined PFAS toxicity in liver tissue from aborted fetuses,^45^ but few other studies in this area are available. The association with heart defects seems to be supported by experimental findings, e.g., the PFOS-associated abnormalities in heart development in a mouse model.^46^

A recent ecological study of women who had given birth in a PFAS-contaminated area in Italy had at least a 50% higher risk of preeclampsia and gestational diabetes.^40^ A systematic review highlighted that the evidence for pre-eclampsia risk was most convincing for PFOA, PFOS and PFHxS, and that weaker support was available for a specific association between PFOS and hypertensive disorders of pregnancy.^47^ Of note, one study found that PFNA had a significantly elevated odds ratio for miscarriage,^20^ while another study reported the strongest association with PFOA.^48^

At least some of these outcomes may well be mediated by endocrine disruption during early development. Hormonal disruptions at elevated PFAS exposures have been detected already in early infancy and childhood.^49,50^ Further, increased PFAS exposure in early life has been linked to changes in sex hormone concentrations both in boys and in girls during or after puberty.^51–53^ Of additional concern, infants with elevated developmental exposure are likely to suffer from impacts on hormonal programming and developmental delays during early adolescence including reduced hormonal output and interference with puberty development in both boys and girls.^52,54–57^ A recent cross-sectional study of girls below 9 years highlighted the positive association between PFAS exposures and serum-estrogen and with early peripheral puberty development.^53^

Duration of breastfeeding also appears to be negatively affected by PFAS-associated endocrine disruption. An initial study of 1400 Danish women reported that the duration of breastfeeding, as recorded by telephone interviews, decreased at increasing serum-concentrations of PFOA and PFOS, although only in multiparous women.^58^ A U.S. study^59^ found that increased maternal serum-PFOA concentrations were correlated with a decreased duration of breastfeeding, and that this association was not confined to multiparous women and was independent of potential confounders. A subsequent study of 1130 new mothers in the Faroe Islands^60^ showed that increased maternal serum PFAS concentrations were associated with a reduction in duration of both total and exclusive breastfeeding, most pronounced for PFOS, where a doubling was associated with a substantial reduction in total breastfeeding of about six weeks. Such effects were also seen for PFOA, though not for PFHxS. Similar results have now been published also from a Danish mother-child cohort^61^ and from an additional U.S. birth cohort^62^. Experimental studies appear to support the epidemiological findings.^63^

### Endocrine disruption

The thyroid gland appears to be a target organ for PFAS exposure, as supported by the C8 panel’s study recognizing the association between elevated PFOA and PFOS exposure and thyroid disruption.^64^ Such associations have also been reported at background exposures,^65^ as also recognized by ATSDR.^1^ Recent studies on thyroid effects have focused on pregnant women and newborns^66–72^ and suggest that thyroid toxicity may begin to occur prenatally and may be detectable also at adolescence.^73^ Because the thyroid gland is the target for a substantial number of other environmentally toxic chemicals, PFAS exposure may contribute to adverse health effects on the thyroid gland from complex exposures, possibly exacerbated by borderline or frank iodine deficiency.^74^

Due to the strong link between thyroxine hormone delivery and early neurological development, disruptions of the thyroid gland may also adversely impact neurological functions. Even a marginal diminution in hormone output is a health concern, as fetal brain development is highly vulnerable to deficiencies in maternal thyroid hormone supplies.^75^ PFASs have been detected in human cerebrospinal fluid^76^ and at low concentrations in brain tissue.^77^

While several studies have pointed to developmental neurotoxicity as a potential effect of prenatal and early postnatal PFAS exposure,^78–83^ it is not yet clear how and to which extent early-life exposure to PFASs affects brain development and if it is sex-dependent.^84,85^ Cognitive deficits are most likely influenced^86^ and may be mediated via reduced attention, as recently suggested.^87^ While cognitive deficits were not identified in other recent studies that considered prenatal PFAS exposure only,^88,89^ another study found weak negative associations, primarily with prenatal exposure to PFOS and PFNA.^90^ A study that relied on maternal pregnancy blood concentrations of PFOA, PFOS, and PFHxS identified cognitive deficits in boys only.^91^ Exposure during infancy was not yet considered in most of these studies.

Associations with risks of autism or ADHD have similarly shown variable results,^92–96^ probably in part due to different study designs and diagnostic criteria. The most comprehensive study relied on 10 NIH-funded prospective cohorts with more than 1,400 children and found these outcomes of neurotoxicity only marginally associated with prenatal exposures, especially for PFNA.^97^ The adverse effects may particularly include hyperactivity and internalizing behaviors.^98,99^ A possible increased occurrence of Alzheimer’s disease and dementia has been reported from exposed Italian communities,^40^ but little evidence is available so far on the possible link between early PFAS exposures and degenerative disease in older adults.

Further, gestational diabetes has been the focus of multiple recent studies,^100–105^ with mostly positive findings. These results are of importance also because gestational diabetes seems to promote the transfer of PFASs across the placenta to the fetus^106^ and may also lead to increased birth size and risk of complications in connection with childbirth. This association was confirmed in a large study from China where adjustment for thyroid function seemed to weaken this association, thus suggesting that thyroid interference might be part of the causative pathway.^107^

In a prospective study of overweight adults in the U.S., elevated PFAS exposure was also linked to an augmented loss of bone mineral, thus suggesting an increased risk of osteoporosis and bone fractures.^108^ In addition, associations have been found between pre- and postnatal PFAS exposures and indicators of childhood and adolescence bone health. One study found an inverse association between prenatal PFOS exposure and bone mass density measured at age 17 years, although the association was null after adjustment for lean body mass.^109^ Another study found inverse associations between prenatal PFOA and PFNA and age-12 bone mineral concentrations in hip and forearm, although not for other bone mass measures.^110^ In a larger cross-sectional study of children at age 8 years, a higher plasma-PFAS concentration was associated with lower bone mass density, with the strongest and most consistent association found for PFOA.^111^ Our recent cohort study at age 9 years found that bone density was negatively associated with PFAS exposures in childhood, i.e., PFDA and PFNA exposures at 18 months and 5 years and PFOA at ages 5 and 9 years, while associations with PFOS were weaker.^112^ Likewise, adolescents and young adults in California showed a negative association between serum-PFOS and bone mineral density.^113^ Although it is not yet clear whether human skeletal development is already adversely affected prenatally, the existing evidence is in agreement with experimental toxicology studies^114,115^ that were considered crucial for risk assessment by ATSDR.^1^

All of these studies document the wide range of abnormalities, many perhaps associated with endocrine disruption, thus suggesting multi-organ toxicities. While possibly hormonally dependent, part of the body weight changes may well be mediated through lipid metabolism in the liver. ATSDR has concluded that PFOA, PFOS, PFNA, and PFDA are associated with increases in serum-lipid concentrations, and such adverse associations have also been documented in children,^116,117^ thus suggesting that elevated lipids in adulthood may be triggered or programmed by developmental PFAS exposures.

### Immune toxicity and cancer

The National Toxicology Program (NTP) recognized both PFOS and PFOA as “presumed” human immunotoxicants, i.e., the level of evidence just below “known”,^118^ as judged from the evidence available in 2016. Subsequent evidence indicates that PFHxS and PFNA are also associated with weakened immune resistance to infectious disease in children.^119^ Immunotoxicity has been the subject of recent reviews,^120,121^ and the likely mechanisms have been explored.^122^ PFAS-induced immunotoxicity has been recognized as the critical effect by major authorities.^16,123^

These conclusions rely in part on studies that show lower concentrations of specific vaccine antibodies in children with elevated exposures to PFASs and an increased risk of not having reached the established clinically protective level, even after three or four vaccinations.^124^ Thus, our first prospective study included 587 children at 7 years of age and showed that a doubling in serum concentrations of PFOS and PFOA was associated with an overall decrease by 50% or more in the children’s vaccine-specific antibody concentrations.^124,125^ EFSA relied on these findings in the most recent risk assessments of major PFASs^39,126^ and also took into account related findings in a smaller study from Germany.^127^ However, in doing so, EFSA decided on tolerable exposure limits for PFOS and PFOA that are too high, due to questionable choices in the calculations.^128^

As the adaptive immune system is programmed during early postnatal development, an immunotoxicity assessment is particularly relevant in subjects with PFAS exposures during early life.^129^ In infants, the major postnatal exposure source is usually human milk,^130,131^ through which a mother may transfer a substantial part of her own body burden of PFASs to the much smaller infant. Obviously, in bottle-fed infants, contaminated drinking water may also cause elevated exposures through formula preparation.^132^ While human milk is considered the ideal nutrition for newborn children, a recent review by the National Academies of Science, Engineering, and Medicine focused on social and other barriers to breastfeeding and ignored the impacts of the chemical contamination of human milk.^133^

Although only one aspect of immune defense against infection, insufficient antibody responses to vaccinations thwarts the desired long-term protection against infectious diseases, likely also beyond the diseases targeted by the individual immunizations. Thus, in a study conducted in Norwegian children, researchers found that increased concentrations of PFOA, PFNA, and PFHxS were linked to statistically significant increases in the frequency of infections such as the common cold and gastroenteritis.^134,135^ Similar findings have been recorded from the U.S. and other countries.^136–141^ and chronic infections appear more common in U.S. adolescents at higher PFAS exposures.^142^ Perhaps more importantly, elevated exposures to PFOS and PFOA are associated with increased hospitalizations for infectious disease in young children, especially for pneumonia,^136^ although that was not apparent when focusing on older children.^143^ A lowered antibody response to vaccines has also been documented in adults,^144,145^ though such associations are not uniformly reported and likely depend on study design.^145^

While there seems to be little association with childhood asthma,^146,147^ PFASs have been found to be linked to certain forms of autoimmune disease. This link was demonstrated by two studies conducted by C8 Science Panel epidemiologists, the first being an occupational study of 3713 workers, whose PFOA exposures were evaluated. Using a ten-year lag, the occurrence of ulcerative colitis and, without a lag, rheumatoid arthritis showed significant associations by greater disease frequencies at elevated PFOA exposures.^148^ A recent case-control study of systemic lupus erythematosus also suggested a possible PFAS-associated risk.^149^ Likewise, association with other disease outcomes that may be affected by immune system dysfunction is possible and is undergoing continuing research.^150–152^

Because the immune system is also crucial in detecting and eliminating cancer cells, immunotoxicity may play a role as a possible mechanism in cancer development.^153,154^ PFASs possess several additional key characteristics linked to carcinogenicity,^155^ and experimental support for the carcinogenicity of PFOA has been published by the National Toxicology Program.^156^ The IARC first concluded in 2016 that PFOA is a possible (Group 2B) human carcinogen,^153^ but updated this in 2023 to Group 1 (human carcinogen), while PFOS was now found to be in Group 2B.^157^ The U.S. EPA considers both PFOA and PFOS as carcinogenic to humans.^38^

An ecological study in California found several childhood cancers related to PFAS-contaminated drinking water.^158^ A recent study reported that prenatal exposure to PFOS and PFOA might increase the risk of retinoblastoma in children.^159^ In adults, data on kidney cancer have focused on the increased risk associated with PFOA exposure,^160^ and substantial evidence has accumulated of PFOA as a cause of cancer of both the kidneys and the testicles.^64,153,161,162^ In addition, available evidence shows that PFASs are also likely to cause an elevated risk of cancer of the prostate,^163–165^ bladder,^166^ breast,^167,168^ and some other sites.^77,169–171^ In a large study carried out by the NCI on breast cancer, there was little evidence of an overall association between PFOS or PFOA exposures and breast cancer risk, but subtype-specific analyses showed clear associations for hormone receptor-positive breast tumors,^172^ a finding that seems meaningful as illustrated by previous research on environmental risk factors in breast cancer.^173^ While colorectal cancer has apparently not been studied in this regard, one study showed that PFAS exposure was linked to an increased number of metastatic lymph nodes, thus suggesting a worsened prognosis of the disease.^174^ The possible increase in metastasis of this cancer in the presence of PFOS and PFOA is supported by in vitro data.^175^ Utilizing data on previous cancer diagnoses, current serum-PFAS analyses showed elevated risks for melanoma and uterine cancer;^176^ although these data may not represent causal associations, they support the notion that PFAS carcinogenicity may not only affect a few particular sites.

## Industry secrecy and delayed public research

Given that DuPont and 3 M were innovators in PFAS production and applications for many years, it is not surprising that much of the early (though limited) exploration of PFAS properties was undertaken or sponsored by these two companies. What does appear to be remarkable is how late the industry knowledge on toxicity and environmental dissemination was publicly disclosed and how little this information inspired the companies and other related industries to conduct in-depth studies to reveal and understand any PFAS-associated risks and the initiation of controls or restrictions to prevent hazardous exposures and environmental dissemination (Table 2). In fact, 3 M researchers documented already in the 1970s the presence of PFOS and PFOA, e.g., in fish.^177^ Knowledge on the risk of PFAS-contamination of local drinking water was particularly obtained at a DuPont production plant early on,^177^ but this discovery apparently did not lead to any intense efforts to understand and control PFAS dissemination in the environment. Industry documents on PFAS production and applications have been collated, e.g., by the UCSF Chemical Industry Documents Library. A recent review of these documents highlights the lack of transparency and the strategies to suppress or manipulate information that could otherwise be disadvantageous to the company.^178^

**Table 2:** Major events or developments in regard to PFAS production, environmental dissemination, and knowledge on toxicity and other properties.^177,178,182,188^

Year	Event	Source
1949	Introduction of Teflon brand	DuPont
1956	Introduction of Scotchgard	3 M
1961	First rat toxicity study	DuPont
1968	Organic fluorine found in human serum	Donald Taves
1978	Monkey study reveals immunotoxicity	3 M
1981	Malformations detected; female workers moved	DuPont
1984	PFOA found in local drinking water	DuPont
1992	Abnormal worker health data	Frank Gilliland
1993	Excretion into milk in experimental goat study	3 M
1998	PFASs detected in human blood bank samples	3 M
2001	PFOS globally disseminated in environment	John Giesy
2003	Falsified article downplays health risks in workers	3 M
2006	Stewardship program: PFOS/PFOA phase-out	U.S.EPA
2020	Proposal to restrict uses of PFASs	EU
2022	Recommendation of close-to-zero exposure limits	U.S.EPA

Table 2 lists important events and discoveries (whether published or not) on the PFASs from their early use, elevated exposure in workers, adverse effects in laboratory animals and in workers, dissemination into the environment, public health, and potential regulatory actions. The first public report that suggested that the general population was exposed to fluorine-substituted organic compounds appeared in Nature in 1968,^179^ where Donald Taves showed that serum proteins contained an elevated amount of non-extractable fluorine that could not be ascribed to inorganic fluoride from drinking water or food. In fact, the Taves’ report followed a previous finding that PFOA was bound to human serum proteins.^180^ Little inspiration seemed to derive from these early findings.

When 3 M started measuring worker blood samples for organic fluorine, it was found that concentrations in exposed workers could be up to 1000-fold higher than the background. At about the same time, a further report from Taves and colleagues showed that there was widespread contamination of human tissues with organofluorine compounds which likely derived from commercial sources such as PFOA (or PFOS).^181^ Health examinations were apparently routinely carried out at 3 M, although it is not clear when formal and systematic records were kept. DuPont and 3 M recorded PFAS in blood from pregnancies and newborns by 1980,^178^ and unusual malformations were then discovered at childbirths among exposed female workers.

Toxicology information was apparently exchanged between the PFAS-producing industries,^182^ although it is unclear how frequently that happened. A joint conclusion of “no adverse human health effects” obviously depended on how hard they looked. Likewise, “no widespread potential for bioaccumulation” is counter to predictions from physicochemical properties, and it is not clear how 3 M justified this optimistic conclusion. Nonetheless, internal 3 M documents show that company scientists had been aware of the health risks of PFOA as early as the 1960s.^183^

Despite being known and in commercial use since about 1950, little was published in scientific journals on PFAS toxicology until the 1980s. Publications on the solubility of PFOA salts were publicly available in the 1960s and were included in a 3 M technical manual. One difficulty was that analytical chemistry could not reliably determine organic fluorides in organic matrices until the late 1970s (particularly at the levels present in the environment).

The 90-day monkey study from 1978 demonstrated toxicity effects on the gastrointestinal tract and the reticuloendothelial system (i.e., the immune system).^184^ The doses of PFOA given were 0, 3, 10, 30 and 100 mg/kg/day. All monkeys at the 100 dosage and three out of four at 30 mg/kg/day died; compound-related microscopic lesions were seen in adrenals, bone marrow (hypocellularity), spleen and lymph nodes (atrophy of lymphoid follicles in both), as also highlighted by Dr. Gilliland in his thesis from 1992, where he added: “No follow-up studies of these observations have been reported”.^185^ Some of the findings were summarized in a published review article from 1980.^186^ A rat study of three PFASs in 1981 showed abnormal eye development in the fetuses, thus suggesting malformations. At Dupont, it was reported that two out of eight child-births among exposed female workers had eye and tear duct malformations.^187^ While women were at first removed from PFOA exposure, a second rat study with somewhat deviating and less worrisome findings led Dupont the following year to conclude that “females of child-bearing age can move back into areas with potential C-8 (PFOA) exposure.”^178^

The first studies on transfer into milk were apparently carried out in 1993, as a document from that time states: “There is some preliminary evidence that in lactating goats PFOS is transferred to milk. It is likely that lactating human females would also transfer PFOS to milk.”^188^ Again, it appears that 3 M decided not to publish the results, and it appears that 3 M did not follow up with analyses of human milk to establish the potential implications of this discovery.

As late as 1998, a 3 M report to the EPA described the widespread occurrence of PFASs in serum samples from blood banks, and environmental contamination and the persistence of the PFASs in the human body therefore became an increasing concern. The most comprehensive mortality study of 3 M workers compared to supposedly unexposed workers from 1947 to 2002 was included in a doctoral thesis by Katherine Raleigh.^165^ By dividing the workers into six different levels of exposures, a dose-dependent risk was apparent for prostate cancer, although not statistically significant. However, the published report^189^ that was co-authored by 3 M colleagues compared with the general population and found an absence of a trend in quartile exposure groups. The discussion section of the published article notes that family history of prostate cancer may play a role. Indeed, another study published the same year^163^ showed an elevated PFAS-associated prostate cancer risk in subjects from the general population who had a family history of the disease.

A subsequent article on worker health by 3 M authors included only selected and edited results without *p* values from four of the multiple tables available in the draft.^190^ The more extensive draft version with entirely different conclusions is accessible, and the published study must be considered as possible scientific dishonesty, as suggested by my letter to the editor of the journal in 2018.^191^ The four-page response from a 3 M author did not provide any clarity or admission of error.^192^ By 2026, the article has not been retracted, but has been cited more than 300 times in the scientific literature and by regulatory agencies, possibly as documentation of lack of toxicity at high levels of occupational PFOA exposures.

An annual report from a production company claimed: “Based on over fifty years of industry experience and extensive scientific study, DuPont believes there is no evidence that PFOA causes any adverse health effects or harms the environment”.^182^ This wording is quite similar to statements from other PFAS industries. An internal document from the 3 M company instructs that the purpose of scientific publication was to show “that there is no medical or scientific basis to attribute any adverse health effects to 3 M products.”^177^ Accordingly, the existence of this serious type of publication bias in the occupational health studies must be considered when assessing the scientific literature. Such examples are well known from other parts of the private sector.^193^

### Growth of scientific recognition

**Fig. 1:**
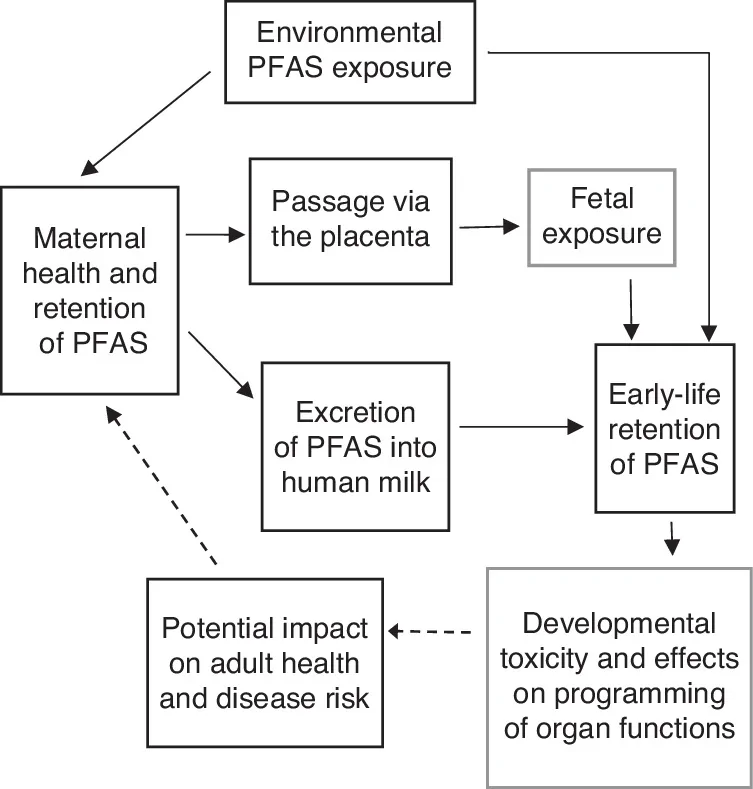
Pathways of early-life exposures to PFASs and possible long-term consequences. Dashed lines indicate hypothetic connections.

Few epidemiology studies have focused on late consequences of PFAS exposures during infancy or prenatally, despite the fact that early development is considered a highly vulnerable period that must be taken into regard when determining exposure limits.^194,195^ It is remarkable that such studies and potential epigenetic effects were apparently not carried out under industry auspices in the several decades of expanding commercial production. Figure 1 illustrates the possible pathway of epigenetic effects where PFAS exposure may cause adverse outcomes in the next generation and beyond.

Since the first reports in scholarly journals on widespread global occurrence of PFOS in wildlife and the detection of PFASs in blood from the general population (about year 2000), the scientific literature on the environmental and toxicological aspects of PFASs has increased substantially, with annual numbers of health-related publications on the PFASs now exceeding 500. When using the National Library of Medicine database (PubMed), the search items ‘perfluorinated’ or ‘perfluoro*’ return more than 30,000 articles for all years, of which about 30% are retained when limiting to ‘human’. Of these, about 1700 are classified as referring to children and 600 to infants, as of 2026. Again, many of these publications relate to medical technology uses, or are merely descriptive, thus only to a limited extent dealing with adverse human health effects from environmental exposures. Only 60 articles were published before 1990 and 179 during the 1990s. Many of the early studies focused on properties of different PFASs and often contained only descriptive information.

Given that most of the published articles on human health risks from PFASs are fairly recent, the toxicological emphasis developed during the most recent years, thus slowly emerging several decades after the first industry discoveries of PFAS toxicity. Also, the reports from the court-mandated C8 studies from about 15 years ago^64^ constitute a fairly recent development and relied mainly on cross-sectional studies of adults, although fortunately on large population groups.

Most of the evidence at hand therefore remains rather recent and is unlikely to represent the full toxicological perspective, such as those outcomes that may occur at a delay, and some adverse effects and vulnerable subpopulations may not yet have been identified and reported. The occurrence of adverse effects following chronic and early-life exposures to slightly elevated PFAS levels still deserves to be extended, especially regarding the long-term effects of developmental exposures. As has been seen on numerous occasions of modern pollutants,^196^ the evidence available today may well underestimate the true extent of the PFAS toxicity.

## Regulatory responses

As a result of mounting concerns both amongst the general public and the scientific community, public health officials have responded by taking measures to address elevated exposures to PFASs, including restricted PFAS concentrations in drinking water, in soil, and in other media. In 2009, the U.S. EPA set a “provisional” health advisory level for PFOA and PFOS in drinking water of 400 ppt (or ng/L). Then in 2016, U.S.EPA significantly lowered the advisory to a combined health advisory level of 70 ppt.^197^ Two years later, the ATSDR identified minimal risk levels that corresponded to approximately 21 ppt and 14 ppt PFOA and PFOS, respectively, in drinking water, and limits of 140 ppt for PFHxS and 21 ppt for PFNA.^198^ An updated profile from 2021 provided even lower exposure limits.^1^

In June 2022, the EPA issued interim health advisories for PFOA and PFOS. Based on immunotoxicity in children^124^ and the need to protect women in reproductive ages.^195^ The EPA used benchmark dose calculations to arrive at chronic Reference Doses of 1.5 pg/kg·day for PFOA and 7.9 pg/kg·day for PFOS; these limits correspond to 0.004 ng/L (ppt) drinking water for PFOA and 0.02 ng/L (ppt) for PFOS,^123^ i.e., substantially below the current limits. Subsequent to the announcement in 2022 of the greatly decreased Reference Doses for PFOA and PFOS, EPA decided that both PFASs were considered possible carcinogens, and the proposed Maximum Contaminant Level Goals would therefore be zero for both compounds.^38^ Enforceable water limits were then proposed at 4 ppt for both PFOS and PFOA, quite similar to limits applied in the EU. For additional PFASs, where human data are much more limited, EPA proposed a Hazard Index, i.e., at 9 ppt for PFHxS and 10 ppt for PFNA in drinking water.

Efforts to combat the harms associated with PFAS exposure are also occurring worldwide and are particularly intensive in Europe. As the PFASs are “known to persist in the environment longer than any other man-made substance”, the EU has announced a proposed plan to eliminate PFAS uses by 2030.^199^

Together, these agency responses indicate a delayed, though rising global concern over the harmful effects of PFASs on human populations and the urgent need to prevent and reduce human exposures to them. Given that science over time has documented adverse health effects at lower and lower background exposure levels and that several health outcomes of relevance have not yet been sufficiently researched, every effort should be made to control adverse health effects in the exposed subjects and to minimize population exposures, especially those occurring at early ages.

## Perspectives

Given the widespread exposures that might not be known to the affected populations, the NASEM recommends clinical testing and follow-up of subjects with likely elevated exposure.^16^ Although supported by ATSDR,^200^ this public health need has generally not been met, probably due to the high costs, e.g., of serum-PFAS analyses. As a result of the unexpected exposures and the growing documentation of PFAS toxicity, a wealth of legal cases has emerged. Although PFAS manufacturers and users have already paid large sums of money in compensation, the PFAS contamination of the global environment remains, and essentially all people have a body burden of PFAS that will remain for many years. And children will continue to be exposed at an early age.

Research in this field has emerged only recently, and the current knowledge of PFAS toxicity is undoubtedly incomplete. One indication of the growth of knowledge is the proposed changes of EPA’s PFAS exposure limits. Over less than two decades, the recommended limits have decreased by more than 1000-fold, a strengthening that is highly unusual. Of particular concern is pediatric toxicity, where PFAS-induced developmental changes appear to indicate the likely progression of important diseases at adult age. Although experimental studies support the existence of delayed adverse effects, epidemiological confirmation is limited due to the difficulty in designing and carrying out such long-term studies extending from birth to adulthood.

Of particular note is the experimental demonstration that PFAS exposure may cause epigenetic changes that are subsequently inherited by future, unexposed generations.^85^ Although such effects have not been reported for PFAS, a recent study suggests that exposure to polychlorinated biphenyls (PCBs) may cause epigenetic effects that result in obesity in humans.^201^ Like PCBs, PFASs are considered obesogens, and epigenetic changes must be regarded a potential risk that deserves attention. This perspective is illustrated in Fig. 1. In the meantime, PFAS exposures will continue.
